# Patient satisfaction with access, affordability and quality of diabetes care at Mohalla Clinics in Delhi, India

**DOI:** 10.3389/fpubh.2023.1160408

**Published:** 2023-04-17

**Authors:** Meenu Grover Sharma, Anu Grover, Kusum Shekhawat, Harvinder Popli

**Affiliations:** ^1^School of Pharmaceutical Sciences, Delhi Pharmaceutical Sciences and Research University, New Delhi, India; ^2^Strategic Scientific Content, Mangrove Creations LLP, New Delhi, India; ^3^Centre for Community Medicine, All India Institute of Medical Sciences, New Delhi, India

**Keywords:** Mohalla clinic, diabetes, primary care, patient satisfaction, India

## Abstract

**Introduction:**

Mohalla Clinics have been set up to provide curative care for minor ailments free of cost within walking distance in the urban slums, thus making primary care more accessible and affordable. Studies evaluating patient satisfaction with treatment of chronic conditions, such as diabetes, in these clinics are lacking.

**Methods:**

A survey of 400 type 2 diabetes patients was conducted, split equally between Mohalla clinics (MC) and Private clinics (PC) in Delhi. Responses were analyzed using STATA17, applying appropriate statistical tests for the data type (Chi-square test, Mann–Whitney *U* test, Wilcoxon signed rank test, or two-sample *t* test).

**Results:**

Satisfaction level was high in both groups with no significant difference between mean satisfaction scores of MC patients and PC patients (Mean 3.79 vs. 3.85 respectively, *p* = 0.4). However, MC patients reported a significant improvement in their satisfaction score after switching to MC (Mean 3.79 vs. 3.3 for the previous facility, *p* < 0.05). Physician interaction with the patients was the most important factor in influencing the satisfaction score. Proximity to the clinic was the second most important factor for MC patients but was not as important for PC patients. Surprisingly, treatment success was considered an important factor for satisfaction level by < 10% MC and < 20% PC patients only, pointing to the need for patient education across both the groups. None of the MC patients mentioned free treatment as a contributory factor to high satisfaction, perhaps because most shifted from a government setup to MC. PC patients had more frequent follow-up visits and blood glucose monitoring, and longer consultation duration compared to MC patients, which were offset by access factors, thus not causing much difference to the satisfaction score between the two groups.

**Conclusion:**

Mohalla clinics are making diabetes treatment accessible and affordable for the marginalized population of Delhi, despite not being designed or fully equipped to care for chronic diseases such as diabetes that require multi-specialty care to monitor and manage multiple co-morbidities and long-term complications. Positive perception of physician interaction and convenient location of the clinics are the two major contributors to the high satisfaction patients expressed with diabetes care at these clinics.

## 1. Introduction

Aam Admi Mohalla Clinics (translated as common man’s neighborhood/community clinics) or simply Mohalla Clinics (MC) were launched by the state government of Delhi in 2015 as a flagship scheme to deliver quality primary healthcare closer to communities, especially the underserved ones such as urban slums (1). These clinics have been established primarily to provide basic curative care for common illnesses like fever, diarrhea, skin problems, respiratory problems etc., first aid for injuries and minor wounds, and referral services (2). Patients can simply walk in for a free physician consultation without a prior appointment. Additionally, over 100 medicines included in the essential drugs list are dispensed free of cost to the patients in the clinic itself and over 200 diagnostic tests are also available free of cost through empaneled diagnostic laboratories. Each clinic is staffed with a physician, a nurse, a pharmacist, and a laboratory technician to provide outpatient services (3). Reduced time in commuting to the clinic, less wait time vs. government hospitals, availability of consultation, drugs and diagnostic tests free of cost and under the same roof are the key benefits of MCs which made these clinics quite popular, and the same model is being adopted for provision of primary care services by some other states of India as well (1–5). However, only a limited role of MCs has been envisioned for preventive services, mostly limited to antenatal and postnatal care and nutritional status assessment and counseling. MCs are also not generally meant for specialist consultation, for which a second tier of facilities in the form of Delhi government multispecialty polyclinics are available (2).

A total of 522 MCs are functional as of December 2022, i.e., 1 clinic per 60,000 as against the initial target of setting up 1 per 20,000 population (4). The clinics operate from Monday through Saturday for 6 h every day and have provided over 18 million OPD consultations in the year 2021–2022 (6), which means approximately 117 patients treated per clinic per day. MC doctor, pharmacist and other staff are paid per-patient basis, thus incentivizing treatment of as many patients as possible, even though per patient fee is rather small at INR 40 (roughly USD 0.5 per patient) for the doctor and INR 12 (USD 0.15 per patient) for the pharmacist currently (6). Marginalized groups such as women, older adults, poor and those with education up to primary school, who generally encounter higher barriers in accessing healthcare form a significant proportion of the MC beneficiaries (7). Patient and prescription records are maintained by the MC staff through government provided tablets and clinic software, however there is no published official report or analysis of patient demography or disease types treated at MCs (6). A recently published survey with 356 community participants reported that fever/cough/cold, thyroid, and body ache are the most common medical complaints for younger patients (0–40 years) seeking treatment at MC, and fever/cough/cold and diabetes among beneficiaries older than 40 years of age (8).

Diabetes is one of the leading causes of burden of disease in India and specifically in Delhi, accounting for 3.2% of total disease adjusted life-years (DALYs) in Delhi in 2016, up from 1.3% in 1990 (9). Prevalence of diabetes in Delhi has been reported to be quite high at 18.3% and a diagnosed prevalence of 10.8% (10), thus representing a significant size of patient population of the state seeking treatment. The goals of treatment in diabetes include glycemic control as well as prevention of microvascular complications such as retinopathy, neuropathy and nephropathy, and macrovascular complications such as cardiovascular, cerebrovascular, and peripheral vascular disease. Thus, contrary to the main aim of MCs of curative treatment of acute conditions, treatment of diabetes requires frequent follow-ups, long-term monitoring of the disease and associated complications, and preventative and promotive health services. Despite not having specialists at MCs, diabetes has been reported as one of the major ailments for patients seeking treatment in these clinics (8). There are only a few studies reporting satisfaction with MCs at a community level (7, 8, 11–13), however, in our knowledge no studies have thus far evaluated satisfaction level of patients with diabetes treatment at these community clinics which are not *per se* designed for management of such chronic conditions with long-term sequelae.

## 2. Methods

The study was conducted using a structured questionnaire-based survey with 400 type 2 diabetes adult patients, split equally between those seeking treatment at MCs and those getting treated at any private clinic (PC) in Delhi. The field survey was conducted between July 2022 and October 2022, using convenience sampling method, with the sample spread out across various zones of Delhi for both MC and PC patients and not concentrated in one or two centers only. The final sample comprised respondents from 19 different MCs and 24 PCs across the city.

### 2.1. Data collection and study tools

Two separate questionnaires were developed-one for MC patients and the other for PC patients. Both the questionnaires included questions related to demographic details, satisfaction level, factors influencing satisfaction score, access factors and cost of treatment. Additionally, MC patients were specifically asked about their previous facility before shifting to MC, satisfaction score for previous treatment center and facilities available in the MC they currently get treatment from. On the other hand, questions related to awareness about MC and barriers to seeking treatment at MC were unique to PC patients’ questionnaire. Before beginning survey data collection, a pilot phase was conducted with 10 patients (not a part of the final analysis), for pre-testing the questionnaires. The final questionnaires were also translated into Hindi (locally spoken language) for convenience of administration to the respondent population. Patients were interviewed in-person while waiting before/exiting after OPD consult at the clinic where they were seeking regular treatment of diabetes. An informed consent was obtained for respondents and anyone who refused to participate was excluded. Each interview took about 15–20 min. The study design was approved by the Ethics Committee of the institute.

### 2.2. Data analysis

Responses were analyzed using spreadsheets (Microsoft Excel) and statistical analysis performed using STATA17. Appropriate statistical tests of significance were used based on the type of data and objective of the analysis. Chi-square test was used for majority of data points for comparative analyses of MC and PC patients’ responses on close-ended questions. Other tests used were Mann–Whitney *U* test (for Likert scale questions on satisfaction score), Wilcoxon Sign Rank test (for before and after comparison for Mohalla clinic patients), and two sample *t* test (for comparison of cost of treatment).

## 3. Results

The basic demographic profile of the study population is summarized in Table 1. The survey groups were largely similar in profile in terms of gender and age distribution. However, those getting treated at PCs were more educated (22.5% of MC patients were illiterate vs. none among PC patients, only 10.5% of MC patients were graduate or above vs. 41.5% of PC patients) and had a much higher household income (over two-thirds of MC patients had income less than INR 10,000 ($125) per month while 80% of those treated at PCs had household income over INR 30,000 ($375) per month).

**Table 1:** Demographic profile of the survey population.

		Mohalla clinics, *n* (%)	Private clinics, *n* (%)
Gender	Male	84 (42%)	105 (47.5%)
	Female	116 (58%)	95 (52.5%)
	Other	0 (0%)	0 (0%)
Age	20–40	77 (38.5%)	84 (42%)
	40–60	89 (44.5%)	94 (47%)
	> 60	34 (17%)	22 (11%)
Education	Illiterate	45 (22.5%)	0 (0%)
	Primary school	42 (21%)	13 (6.5%)
	High school	92 (46%)	104 (52%)
	Graduate	21 (10.5%)	80 (40%)
	Postgraduate	0 (0%)	3 (1.5%)
Monthly household income (INR)	<10 K (<$125)	135 (67.5%)	0 (0%)
	10-30 K ($125–$375)	59 (29.5)	38 (19%)
	30-50 K ($375–$625)	6 (3%)	143 (71.5%)
	>50 K > $625	0 (0%)	19 (9.5%)

The majority of patients getting treatment at MCs either started their diabetes treatment at MC only (34.5%) or were being treated at a government dispensary (27%) or a government hospital (16%), with only 22.5% seeking treatment at a PC earlier.

### 3.1. Satisfaction with treatment

**Figure 1:**
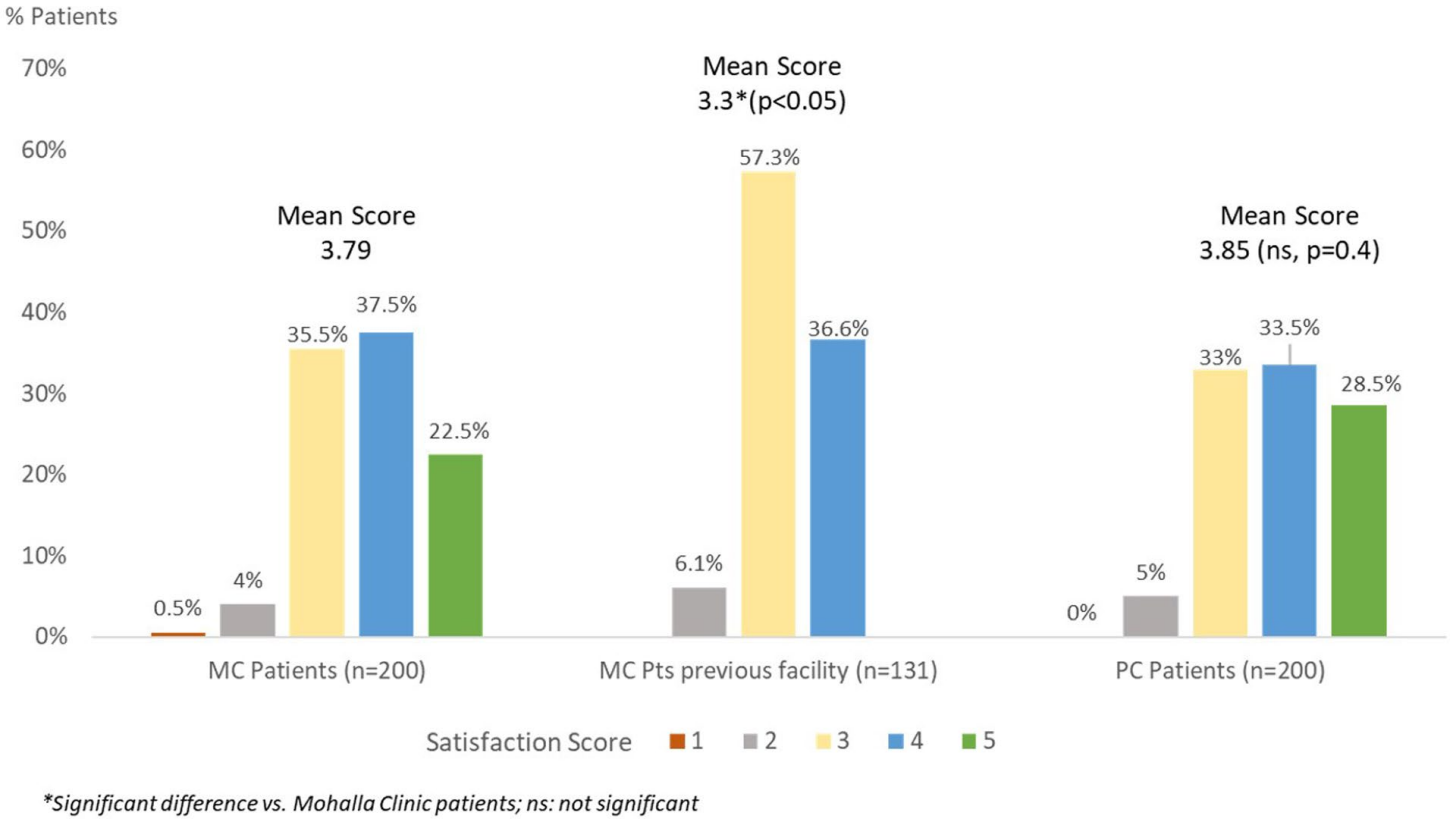
Overall satisfaction score of Mohalla clinic and private clinic patients with diabetes treatment.

Both the groups of patients generally demonstrated a high level of satisfaction with their treatment (Figure 1), with 60% of those being treated at MCs indicating a score of 4 or 5 on a Likert scale of 1 to 5, with 5 being extremely satisfied. The corresponding number for patients undergoing treatment at PCs was 62%. No significant difference in satisfaction score was observed between MC (Mean score 3.79) and PC (Mean score 3.85) patients (*p* = 0.4, Mann–Whitney *U* test). On the other hand, those who had switched to MC from other treatment centers demonstrated significantly better satisfaction score for MC vs. their earlier treatment centers (Mean score 3.3; *p* < 0.05, Wilcoxon sign rank test for before and after comparison).

### 3.2. Factors affecting satisfaction score

**Figure 2:**
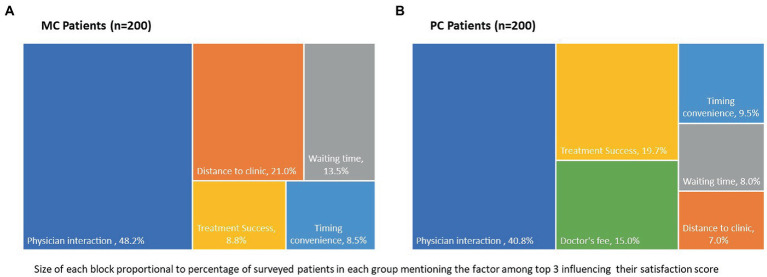
Top 3 factors influencing satisfaction with diabetes treatment for **(A)** Mohalla clinic patients and **(B)** private clinic patients.

Patients were asked to select the top 3 factors that influenced their satisfaction rating with the treatment. Although there was a significant difference in relative importance of various factors (Chi-square test, *p* < 0.05) between MC and PC patients, there were similarities in some factors chosen by both the groups. Physician interaction (spends adequate time with me, listens to me patiently, explains about the disease and how to take medicines) came up as the most important factor in satisfaction of patients with their treatment with 48.2% of Mohalla patients and 40.8% of PC patients ranking it as one of the top 3 factors influencing their satisfaction levels (Figure 2). Distance to the clinic was the second most important factor for MC patients with about 19% mentioning proximity to the clinic as one of the 3 most important factors for high satisfactions levels. On the other hand, distance to clinic was considered an important factor only by 7% of PC patients (Chi-square test, *p* < 0.05). Surprisingly, treatment success (my diabetes is under control) was considered one of the top 3 factors for satisfaction by only a minority of patients, more among the PC patients 19.7 vs. 8.8% of MC patients. Treatment cost/doctor’s fee was another factor showing significant difference as a contributor to satisfaction level between MC and PC patients (Chi-square test, *p* < 0.05). Remarkably, none of the MC patients mentioned free treatment as a contributory factor to high satisfaction, perhaps because most shifted from government setup to MC. On the other hand, 15% of PC patients considered doctor’s fee as one of the top 3 factors. There was no significant difference between timing convenience as a factor for satisfaction score (Chi-square test, *p* = 0.5).

### 3.3. Access factors

**Figure 3:**
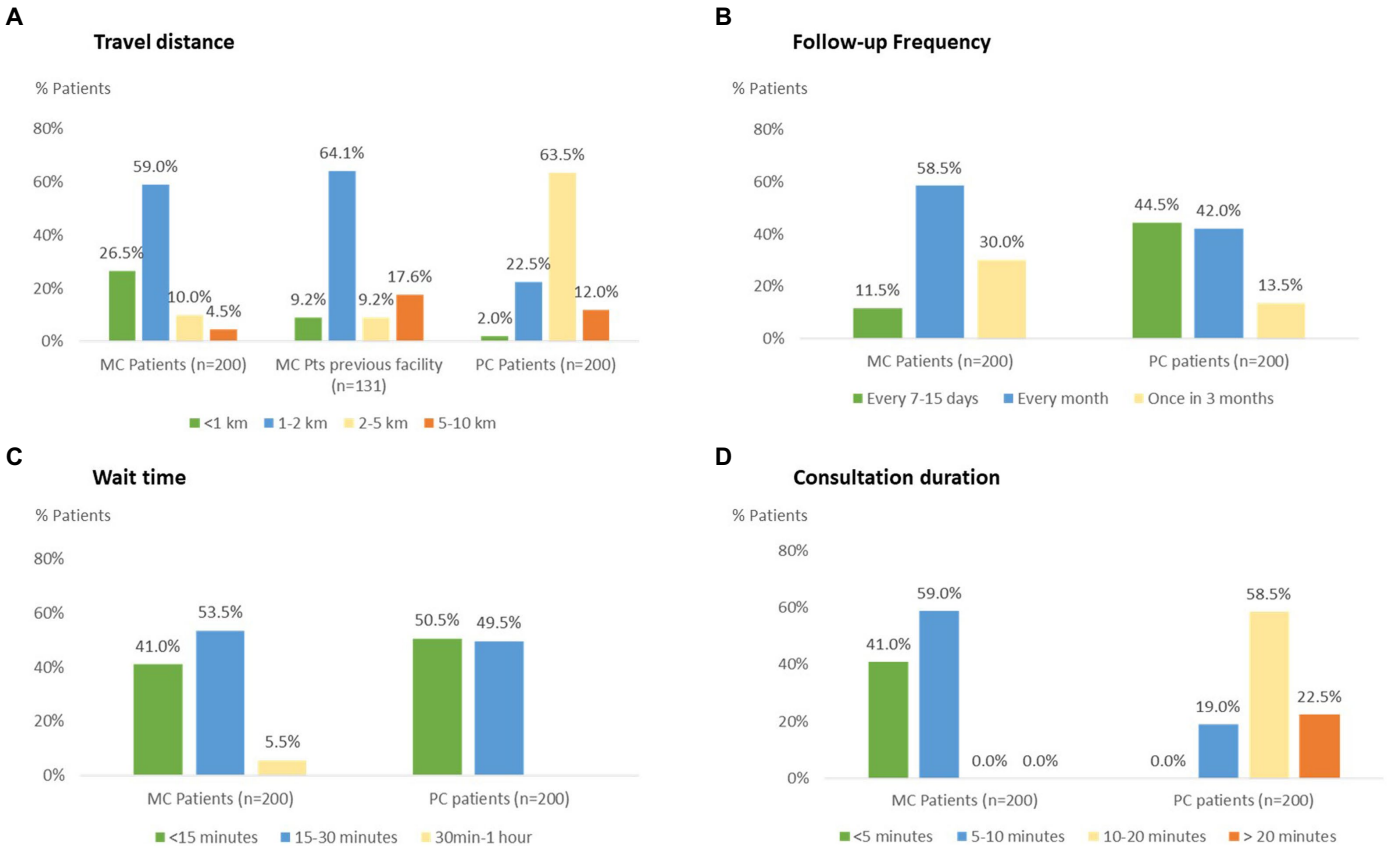
Comparison of access factors for Mohalla clinic and private clinic patients: **(A)** travel distance **(B)** follow-up frequency **(C)** wait time for consultation, and **(D)** consultation duration.

Patients were specifically asked about distance to the clinic, ease of getting appointment, waiting time and consultation duration that contribute to convenience and access to treatment (Figure 3). There was a significant difference (Chi-square test, *p* < 0.05) between MC patients and PC patients on the access factors tested, with MC patients having a favorable situation in terms of proximity to the clinic and ability to walk in without prior appointment but PC patients having a better position in terms of frequency of consultations and time spent with the physician. More than 85% of MC patients travelled less than 2 km to reach their treatment facility. Travel distance for PC patients, on the other hand, was longer with about 76% needing to travel 2 kilometers or more. Further, 70% of MC patients and 86.5% of PC patients have a follow-up consultation at least once a month for diabetes treatment, but significantly more PC patients (44.5%) have a follow-up consultation at least once in 15 days vs. only 11.5% MC patients. While all MC consults are on walk-in, 44% of PC patients need to book a prior appointment for meeting their treating physician. Despite prior appointments, PC patients indicated similar wait time for consultation as the MC walk-in patients. However, MC patients indicated shorter consultation duration, with 80% of PC patients spending 10 min or more with their doctor but none of the MC patients get >10 min with their treating physician.

### 3.4. Facilities available at Mohalla clinic

Patients at MC are provided with free medicines and diagnostic tests that are prescribed by the MC physician. On being asked about the availability of these services, 25.5% indicated availability of all medicines on every visit while another 8.5% mentioned getting all medicines on repeat visit. 55% of MC respondents indicated that some medicines have to be purchased from the private market. Only 11% indicated not receiving medicines they needed. For diagnostic tests, more than half stated consistent availability of diagnostic tests on every visit and 31% shared the need to get some of the tests from the private market.

### 3.5. Treatment cost

**Figure 4:**
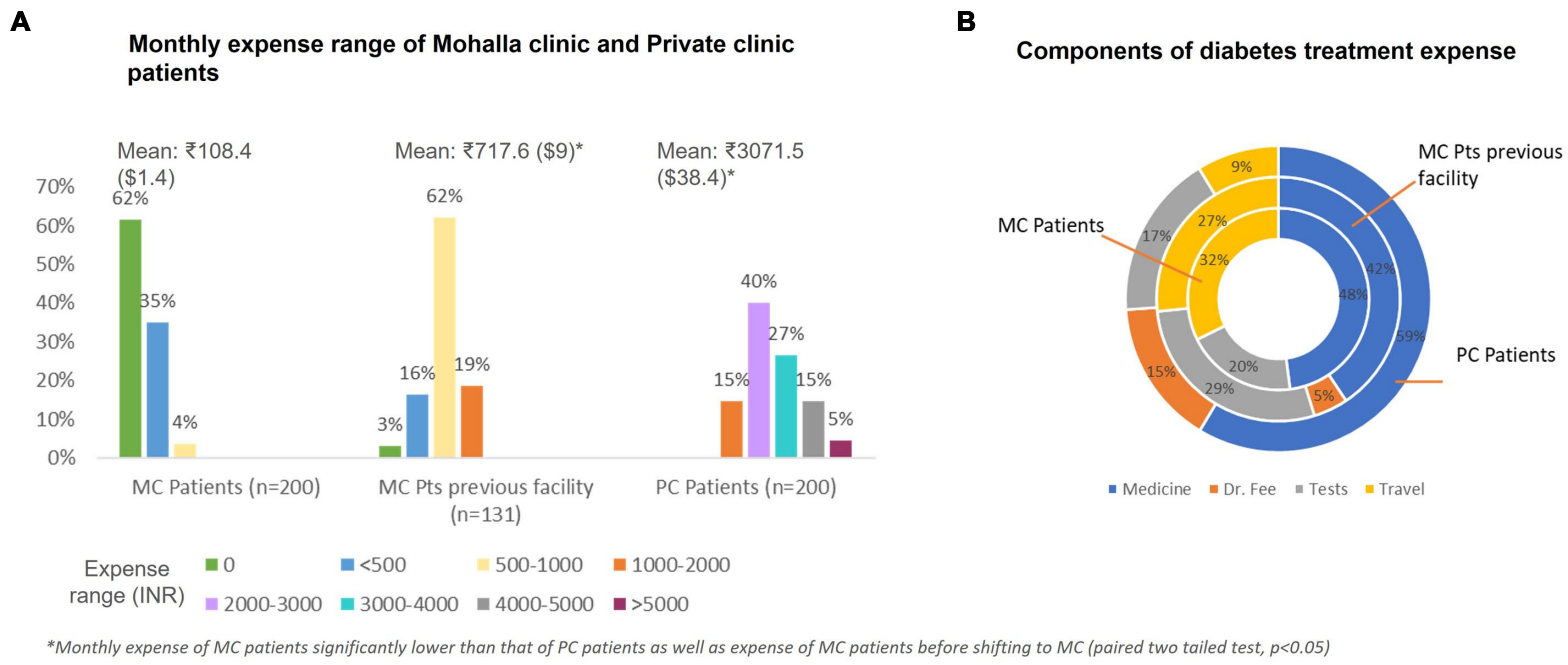
Diabetes treatment expense for Mohalla clinic and private clinic patients **(A)** monthly expense range and **(B)** components of treatment expense.

Total monthly cost of treatment for MC patients were expectedly low (Mean: INR 108.4, SD: 173.3) which was much lower than the cost these patients had to bear (Mean: INR 717.6, SD: 338.5) before shifting to MC and also significantly lower than the PC patients (Mean: INR 3071.5, SD:982.0). The major contributor to the cost in all groups was for medicines. Expectedly, many MC patients (62%) have zero expense, while before moving to MC only 3% had no expense on diabetes treatment (Figure 4).

### 3.6. Glycemic control

Patients were asked about frequency of getting fasting blood glucose (FBG) checked and their last FBG value. PC patients had more frequent monitoring of FBG in comparison to MC patients (Chi-square test, *p* < 0.05). Based on self-reported most recent FBG level, almost all surveyed patients had levels above the desired level of ≤100 mg/dl in both the groups. However, correlation with disease duration, treatment adherence and comorbidities was not made, hence clinical significance of this difference cannot be ascertained.

### 3.7. Barriers to seeking treatment at Mohalla clinic

**Figure 5:**
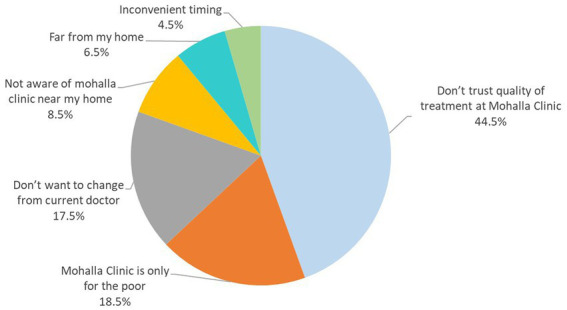
Barriers to seeking treatment at Mohalla clinic (Private clinic patients, *n* = 200).

Private clinic patients were asked about awareness about MCs and why they do not seek treatment at the free, government provided MC facility. All PC patients were aware of MCs, 60% indicated having an MC near their home, but none of the PC patients had ever tried getting treatment for any disease there. A significant percentage carried the bias that quality of care offered there would be sub-par, and some believed that a government run clinic was meant only for the poor (Figure 5).

## 4. Discussion

Healthcare services in India are provided both by the public sector and private players, with a significant skew towards the private sector. Private facilities provide healthcare access for about 70% of outpatient visits and 60% of hospital admissions (14), with an even greater bias in urban areas where 79% of outpatient visits are serviced by the private sector (15). However, in the absence of any financial protection or insurance coverage for outpatient treatment costs in India, all such expenses must be borne out-of-pocket by the patients, making private facilities unaffordable for the poor. Public facilities in India are available free of cost but are overburdened and are straddled with deficiencies and inefficiencies of infrastructure. Further, referral pathways have not been established leading to overcrowding of secondary and tertiary health facilities in the public sector as patients can directly go to these hospitals for any disease type/severity. MCs were instituted in Delhi with an aim to decongest hospitals and offer the convenience of treatment access for minor ailments close to the lower socioeconomic neighborhoods. These community clinics have provided over 50 million consultations in the last five years (6), thus easing some of the patient burden on hospitals.

Studies conducted to evaluate the utilization and performance of MCs have reported high rates of satisfaction among the neighborhood community that accesses these clinics. A recent review reported a generally high level of satisfaction (~ 90%) with the MC services, which were considered either at par with or better than other existing healthcare facilities the patients accessed earlier (7, 8, 11–13). In a recently published community survey, MC users expressed a high level of satisfaction with the MC doctor and gave an average rating of 4.1 out of five (8). In another study, most patients indicated a high intent to return to the MC for seeking care in the future (5). While obvious factors such as proximity to the place of residence, shorter wait time vs. government hospitals, interaction time with the doctor and effectiveness of treatment have been cited as the reasons influencing decision of patients to return to MCs for seeking care, the most important factor suggested in this study is the interaction time with the doctor and other healthcare providers that can play a pivotal role in ensuring success of such initiatives (5).

Our study also pointed to physician interaction as the number one factor influencing satisfaction level of the patients attending MCs as well as those seeking care at private settings with 44.5% of all surveyed patients ranking it as one of the top 3 factors. A doctor who listens patiently and is perceived as providing adequate advice on management of their condition is the critical factor all patients are looking for while evaluating their treatment. MC doctors treat an average of 117 patients every day (6 h) (6) which means roughly 3 min on average per patient. In our survey also, 41% of MC patients said they get less than 5 min with the doctor while 80% of PC patients get more than 10 min with their doctors at each visit. The shorter consultation duration of MC patients appears to have been offset by the proximity of the clinic and the quality of physician interaction, translating into high satisfaction levels. Lower expectations of the marginalized population that these clinics serve may be another reason for ignoring the short consultation duration while indicating satisfaction level. A significant proportion of these patients switched from government dispensaries (another form of primary care set up) or government hospitals to MC and hence they may be used to overcrowding and being rushed through the appointment. In fact, those that shifted from government hospitals showed an improvement in satisfaction score from a mean of 3.4 at earlier hospital to 3.6 at MC and those who were receiving care at a government dispensary earlier showed a significant improvement in satisfaction score from a mean of 3.2 at the dispensary to 3.8 at MC. The major reasons cited for improvement in satisfaction after shifting were proximity to the facility and easy availability of free medicines and diagnostic tests vs. the earlier government facility.

A surprising finding in our survey was the lower value both the groups of patients placed on diabetes control (treatment success) relative to other softer aspects like physician interaction or convenience factors like distance and wait time, in choosing the top 3 factors for satisfaction. Less than 10% MC patients and less than 20% PC patients indicated treatment success as a factor influencing their satisfaction score, which may be a reflection of inadequate awareness of the importance of glycemic control. This may also have a correlation with the high self-reported FBG levels at last testing by both the groups. Despite frequent blood glucose monitoring (at least once a month for 80% MC patients and all PC patients) and follow-up appointments (at least once a month for 70% MC patients and over 85% PC patients), self-reported FBG levels were above the normoglycemic range for almost all patients and were also in the very high range for a sizeable survey population. We could not authenticate these self-reported FBG values by comparing them with test reports, nor was our survey designed to correlate these with disease severity, treatment type, lifestyle modification counseling, or treatment adherence related factors. More than one-thirds of MC patients in our survey indicated they started treatment at MC only and have not changed treatment facility in the last 2 years, which means they may not have had a specialist consult even while many of them indicated inadequate glycemic control.

Only a few studies have evaluated patient satisfaction with diabetes care in various healthcare settings in India. A study conducted in urban Puducherry, South India reported high satisfaction levels of about 70% with the health care services received and there was no significant difference in the level of satisfaction between government and private health facility (16). In another study conducted at two centers in the sub-Himalayan region of North India, 70% of patients indicated they were moderately (14%) or highly satisfied (56%) with diabetes treatment, with no significant difference by treatment setting (17). Our survey results indicating high satisfaction level with both the MC (public setting) and the private setting are consistent with the findings in these earlier studies.

**Limitations of the study**: Our study was designed to understand the satisfaction of chronic disease patients with care at MC, taking diabetes as the representative disease. The finding of high self-reported FBG levels in both the groups was surprising but could not be validated with actual test reports. Further, our study was based on convenience sampling and the sample is not large enough for generalization of this finding, neither was our survey designed to correlate high self-reported FBG with disease-related, treatment or counseling-related, patient-related or treatment adherence-related factors. Further studies are recommended to specifically assess the success of diabetes treatment in MCs using actual test reports and/or performing the tests as part of the study. This study was also not designed to evaluate the continuum of care and referral practices for diabetes (or other chronic diseases) for specialist consultation, which can be a part of future studies assessing treatment practices and treatment success. Our study focused on patient experiences and perception only, future studies can combine physicians’ perspective on treatment practices as well. The study also did not aim to identify differences in patient satisfaction or access factors by location within the city, which could also be a subject of future studies.

## 5. Conclusion

The marginalized population of Delhi trusts MCs for seeking treatment of not only minor ailments but also for chronic conditions such as diabetes that require long term follow-up, repeated visits, and frequent monitoring. High patient satisfaction with diabetes care at MCs is backed by an overall favorable perception of physician interaction and proximity of the clinics. Better and more consistent availability of medicines, increased consultation time and periodic consultation with specialists is recommended for further improving the quality of care and patient satisfaction at MCs. Of note, the MC essential drugs list includes only three diabetes drugs (glibenclamide, glimepiride and metformin) and only two drugs for hypertension (enalapril and amlodipine, apart from diuretics) (4), which may be widened to enable MCs to comprehensively manage diabetes patients. MCs collect all patient and treatment related data through clinic software, which can be used to analyze the pattern of care for such patients and to check if there are any missed opportunities to refer to specialist care. Based on the analysis, the state government may consider redesigning/strengthening polyclinics and next tier facilities by adding useful features similar to MCs such as location convenience and easy availability of medicines and tests. Consideration also needs to be given for standardizing protocols for chronic conditions such as hypertension and diabetes for initiating treatment and periodic consultation by specialists to ensure patients get adequate care for managing complications, while regular follow-up and continuation of prescription can be done at the MC. The MCs can also play an important role in preventive screening and counseling in the community for high disease burden chronic conditions in India such as hypertension and diabetes, thus fulfilling the more comprehensive role envisaged for a primary health care facility. Further studies are recommended to evaluate the quality of diabetes treatment at MCs including patient counseling for lifestyle modification, referral practices, treatment success in terms of glycemic control and monitoring of long-term complications, which can further guide standardization of diabetes treatment protocols at the MCs.

## Data availability statement

The raw data supporting the conclusions of this article will be made available by the authors, without undue reservation.

## Ethics statement

The studies involving human participants were reviewed and approved by DPSRU-Biomedical Research Human Ethics Committee. The patients/participants provided their written informed consent to participate in this study.

## Author contributions

MGS conceptualized and designed the survey and the questionnaire, participated in conduct of the survey, data analysis and manuscript preparation. AG contributed to conduct of the survey, data acquisition, and manuscript preparation. KS assisted in data analysis and statistical analysis. HP guided the study concept, protocol design, questionnaire finalization, and reviewed the manuscript. All authors contributed to the article and approved the submitted version.

## Conflict of interest

AG is employed by Mangrove Creations LLP.

The remaining authors declare that the research was conducted in the absence of any commercial or financial relationships that could be construed as a potential conflict of interest.

## Publisher’s note

All claims expressed in this article are solely those of the authors and do not necessarily represent those of their affiliated organizations, or those of the publisher, the editors and the reviewers. Any product that may be evaluated in this article, or claim that may be made by its manufacturer, is not guaranteed or endorsed by the publisher.
